# Gene Flow and Abundance of a Tropical Fruit Fly in a Horticultural Landscape Mosaic in Eastern Australia Is Limited by Cleared Grazing Land and Area‐Wide Management

**DOI:** 10.1111/eva.70097

**Published:** 2025-04-10

**Authors:** James L. Ryan, Anthony R. Clarke, Alexander M. Piper, Susan Fuller, Peter J. Prentis

**Affiliations:** ^1^ School of Biology and Environmental Science Queensland University of Technology Brisbane Queensland Australia; ^2^ Centre for Agriculture and the Bioeconomy Queensland University of Technology Brisbane Queensland Australia; ^3^ Agriculture Victoria Research, AgriBio Centre Bundoora Victoria Australia

**Keywords:** area‐wide management, *Bactrocera tryoni*, effective population size, fruit fly, landscape ecology, landscape genetics, linear mixed modelling, Queensland fruit fly, SNPs, Tephritidae

## Abstract

Landscape ecology and genetics provide important analytical frameworks for investigating the effect of environmental features on ecological processes. Few empirical studies, however, have simultaneously tested how landscape characteristics influence spatial patterns of gene flow and abundance of pest insects in heterogeneous environments. To address this, we undertook a combined landscape‐ecology/landscape‐genetic study of the tephritid fruit fly, *Bactrocera tryoni* , in the Wide Bay‐Burnett region of Southeast Queensland, Australia. This region contains areas of rainforest, *Eucalyptus* forest, cleared pasture, residential areas, and two areas of intensive horticulture production; one implementing area‐wide management practices. We collected *B. tryoni* samples from 26 sites in 2021 during the months of April, August, October, December and the following year during February and April. We used high‐density DArTseq SNP genotyping on samples collected during the 2021 April, August and December sampling periods. We then modelled the contemporary landscape characteristics and management factors influencing gene flow and abundance of this pest species. Genome‐wide SNP analysis estimated infinite effective population sizes at all sites and detected limited genetic structure across the landscape. However, fly abundance varied significantly among habitats, with cleared pasture negatively associated with population abundance and acting as a barrier to gene flow. Additionally, highways in composite with cleared pasture exhibited a very strong barrier effect. Abundance was highest in residential areas and rainforest, lowest in *Eucalyptus* forest, and reduced in the horticultural region with area‐wide management implemented. We discuss the benefits of collecting simultaneous genetic and ecological datasets for informing and evaluating area‐wide management programmes for insect pests and highlight considerations in the spatial analysis of SNP data when effective population sizes are extremely large.

## 1 Introduction

Many of the world's most important agricultural pests are both mobile and polyphagous, meaning that they can breed in noncrop sources before migrating and proliferating in the crop—making on‐farm management alone insufficient (Walter 2003). To counter this problem of invasion from noncrop areas, area‐wide management (AWM) can be utilised (Vreysen et al. 2007). This approach targets pest population suppression across an entire landscape, rather than trying to manage the problem at the level of an individual farm (Hendrichs et al. 2007). Information on the preferred habitat(s) of the pest within the landscape, and how and when the pest moves (or not) across the landscape and between habitat patches is considered core knowledge for the application of this approach (Byrne 2008).

Because they are both polyphagous and mobile, tephritid fruit flies (Diptera: Tephritidae) are frequent targets of AWM programmes (Hendrichs et al. 2002; Vargas et al. 2008; Aluja et al. 2012; Rashid et al. 2021). Knowledge of where tephritids occur in the landscape can come from both adult trapping and fruit collection for larvae (Vargas et al. 1990; Kounatidis et al. 2008; Ortega et al. 2021) or from the release and recapture of marked individuals (MacFarlane et al. 1987; Iwaizumi and Shiga 1989; Peck and McQuate 2004; Froerer et al. 2010). Such studies tend to show that populations build up where breeding hosts are available (Vargas et al. 1983; Paredes et al. 2022) and that while individual adult movement is typically restricted to several hundreds of metres, long‐range dispersal greater than 10 km is not uncommon (MacFarlane et al. 1987; Froerer et al. 2010). However, such studies typically focus on the end‐point of dispersal, and the importance of landscape attributes such as cleared land, waterways, closed forest, etc. in shaping tephritid dispersal patterns remains largely inferred rather than directly tested.

The Queensland fruit fly, *Bactrocera tryoni* (Froggatt), is Australia's most important horticultural pest insect (Dominiak 2019). Research on population suppression or eradication of *B. tryoni* at the landscape level began 60 years ago (Monro 1960; Bateman et al. 1966) and has continued through to the present (Jessup et al. 2007; Lloyd et al. 2010; Tam et al. 2020; Dominiak et al. 2022). Other studies have used expert elicitation in order to rank habitat suitability (van Klinken et al. 2019) or used landscape modelling to make theoretical predictions about random or perfect foraging of *B. tryoni* movement in virtual landscapes (Schwarzmueller et al. 2019). However, despite this extensive research effort, empirical studies on the landscape ecology of *B. tryoni* are lacking (Clarke et al. 2011). Furthermore, none of the existing studies have taken into account genetic relatedness, which reflects the density and dispersal of individuals across the landscape (Wright 1943). Exploiting genetic information and population genetics theory can overcome the limitations of traditional pest monitoring (Beaurepaire et al. 2024) by directly tracking dispersal patterns among putative populations and assessing the influence of landscape variables on observed dispersal patterns without a priori information (Baguette et al. 2013).

To address these knowledge gaps, we carried out a combined landscape‐ecology/landscape‐genetic study of *B. tryoni* in the Wide Bay‐Burnett region of Southeast Queensland, part of the species' endemic range. The region, covering an area of approximately 19,800 km^2^, contains areas of rainforest, which is assumed to be the endemic habitat of *Bactrocera* fruit flies (Drew 1989), as well as eucalypt forest, agricultural grazing land and two areas of intensive horticulture production (the districts of Bundaberg and Gayndah‐Mundubbera). Gayndah‐Mundubbera has exercised area‐wide control of *B. tryoni* since the early 2000s (Lloyd et al. 2007, 2010), while growers in Bundaberg manage fruit flies on‐farm in an *ad‐hoc* manner (Missenden 2014; Senior 2016). In this paper, we report the findings of a year‐long study of adult *B. tryoni* trapped every 2 months from a spatially structured array of sites. We examined the abundance of *B. tryoni* with respect to land use types, or the distance from AWM. Additionally, we examined the influence of land use types on fine‐scale spatial and temporal patterns of genetic structure in *B. tryoni* using genome‐wide single nucleotide polymorphism (SNP) data at three sampling times (April, August, December) corresponding to the end, beginning, and middle of the species' annual breeding cycle. *Bactrocera tryoni* populations decline greatly between April and August during a possible senescence period but then rapidly build again to December before slowly declining through to May (Clarke et al. 2022). Choosing April, August, and December samples for genetic analysis was done based on a priori assumption that these 3 months would be associated with maximum genetic variation within and between different populations within the landscape. Landscape relatedness methods and isolation by resistance (IBR) modelling were used to understand whether land use types act as barriers or facilitators of dispersal. Estimates of effective population size were also calculated for each site. To our knowledge, this is the first combined landscape genetic and ecology study of a tephritid species.

## 2 Methods

### 2.1 Sampling

**FIGURE 1:**
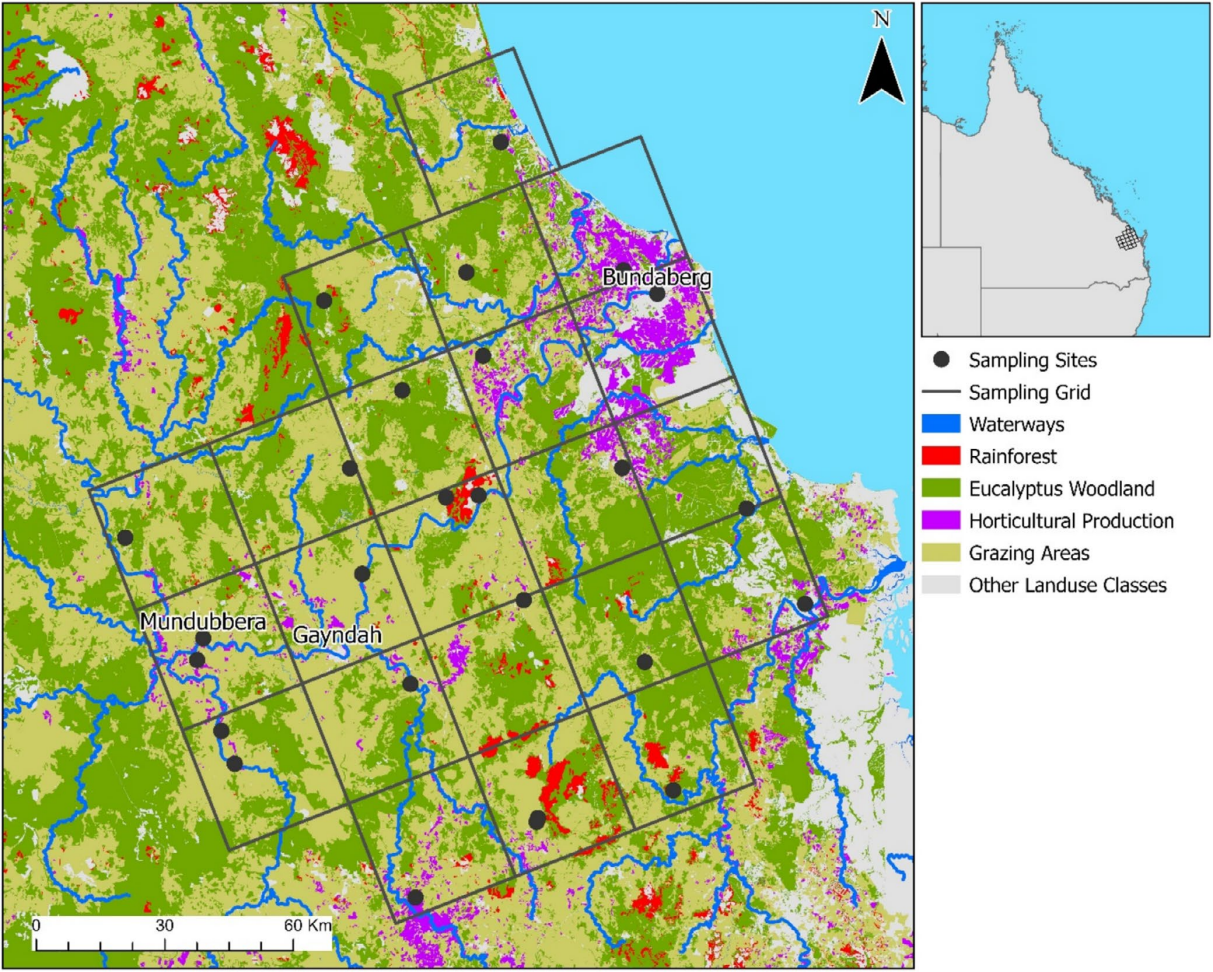
Sampling grid and 26 sampling sites of Queensland fruit fly, *Bactrocera tryoni* , in the Wide Bay/Burnett Region of Queensland, Australia. Included are important land classes in the study area; including rainforest, *Eucalyptus* woodland, horticultural production areas, grazing areas and major waterways. Note, for the Bundaberg district the majority of area identified as ‘Horticultural Production’ includes sugar cane a nonfruit fly host. Approximately 17,000 ha of sugarcane is grown in the Bundaberg region (Rahman and Robson 2020; https://baffa.org.au/about/industries/fibre [accessed 2/08/2024]).

*Bactrocera tryoni* samples were collected from 26 sites across the Wide‐Bay/Burnett region of Queensland, Australia (Figure 1) in April 2021, August 2021, October 2021, December 2021, February 2022, and April 2022. This area was chosen as it has a wide range of land use types, allowing replicated sampling. Furthermore, this area covers two distinct horticultural production areas: Mundubbera/Gayndah and Bundaberg. The Mundubbera/Gayndah production area has a permanent areawide fruit fly management programme, while management in the Bundaberg production area is done on a farm‐by‐farm basis. To have sampling sites distributed throughout the study area, we used a gridded structure for sampling, with each grid square being 30 km × 30 km. Our sampling design consisted of 22 grid squares, with one sampling location chosen within each square, with an additional sampling location in four of the 22 grid squares to sample continuously within a land use type. Samples were collected from five agricultural areas, seven *Eucalyptus* woodland areas, six rainforest areas, and eight residential areas. Commercially sourced male cue lure fruit fly traps (https://bugsforbugs.com.au/product/fruit‐fly‐trap/) were hung from between seven to 14 days (the same number of days within a sampling time period, different number of days between sampling time periods) to catch *B. tryoni* at each sampling location. Samples were preserved in propylene glycol while in the traps and then transferred to 100% ethanol and stored at −20°C. Samples were identified according to the diagnostic features described in The Australian Handbook for the Identification of Fruit Flies v3.1 (Plant Health Australia 2018).

### 2.2 DNA Extraction and Genotyping

DNA was extracted from 545 male *B. tryoni* samples (188 samples from April 2021, 182 samples from August 2021 and 175 samples from December 2021) using the Qiagen DNeasy Blood and Tissue kit according to the manufacturer's protocol. The final elution step was modified by preheating buffer AE to 60°C and incubating the buffer on the spin column for 5mins before final spin down. Extracted DNA was checked for quality and concentration on agarose gels stained with gel red and using a nanodrop spectrophotometer. DNA was sent to Diversity Arrays Technology (DArT) for DArTseq high‐density genotyping. The restriction enzyme combination used by DArT is PstI/SphI. SNPs were called based on a proprietary DArT pipeline (Tomkowiak et al. 2022) and mapped to the *B. tryoni* reference genome, CSIRO_BtryS06_freeze2 (available at NCBI under BioProject number PRJNA560467).

### 2.3 Population Genetics

The dartR package v2.7.2 (Gruber et al. 2018; Mijangos et al. 2022) was used to filter SNPs, retaining biallelic sites with a call rate of > 95% and a minor allele frequency > 0.01. Additionally, we filtered SNPs that deviated from Hardy–Weinberg equilibrium (HWE) expectations within each population separately as this has been shown to detect population structure more reliably than alternative HWE filtration methods (Pearman et al. 2022). Additionally, we used dartR to estimate site‐specific population genetic parameters at each sampling time including observed SNP heterozygosity (H_O_), expected SNP heterozygosity (H_E_), inbreeding coefficient (F_IS_) and conduct a principal coordinates analysis of individual genetic differentiation (PCoA). We used discriminant analysis of principal components (DAPC), implemented in the R package adegenet v2.1.3 (Jombart 2008; Jombart et al. 2010) to estimate the number of populations in our data using values of *K* from 1 to 20. The optimal value for *K* was estimated using the Bayesian Information Criterion (BIC). We also used fastStructure (Raj et al. 2014) to independently assess the number of clusters using a range of *K* = 1–10. We used the chooseK.py utility programme provided by fastStructure to determine the optimal value for *K*. Barplots for different cluster scenarios were generated using pophelper v2.3.1 (Francis 2017) in R (R Core Team 2022). We used the R package SNPRelate v1.32.2 (Zheng et al. 2012; Zheng et al. 2017) to calculate pairwise kinship values between individuals using the maximum likelihood estimation method and 1000 permutations. For downstream analysis, we then calculated the mean kinship for all pairwise comparisons between sampling sites and excluded all pairwise comparisons between sites that had fewer than five samples. A Mantel test was used to examine patterns of isolation by distance for each sampling time period, using mean kinship (transformed to 1—mean kinship) as the measurement of genetic dissimilarity. An AMOVA was conducted using the R package poppr v2.9.3 (Kamvar et al. 2014, 2015) to estimate the genetic variation contained within and among populations using kinship (transformed to 1—kinship) as the distance variable. Effective population size (N_e_) was estimated using the linkage disequilibrium method implemented in NeEstimator V2 (Do et al. 2013) for each sampling site at each sampling time.

### 2.4 Landscape Genetics

Eleven resistance surfaces (RS) were developed in ArcGIS Pro v2.9.1 using landscape predictors which were considered to have the potential to affect *B. tryoni* movement (Clarke et al. 2011; Clarke 2019; van Klinken et al. 2019), and hence gene flow, across the study area. The ‘Minimum Bounding Geometry (Data Management)’ geoprocessing tool was used to define the shape for each RS, and a 1 km buffer was applied to restrict the number of pathways RS modelling may include in the analysis. Because IBR analyses incorporate all possible pathways between pairwise comparisons (McRae 2006; McRae et al. 2008) clipping the extents of each RS was done to exclude biologically unreasonable dispersal pathways during optimisation (Cameron et al. 2019). We developed eight categorical binary RSs representing presence/absence of horticultural areas, highways, grazing areas, minor roads and tracks, rainforest areas, *Eucalyptus* woodlands, waterways and residential areas. Each RS was developed by rasterising vector data retrieved from Queensland Spatial catalogue—QSpatial (see Appendix S1–S14 for spatial layer metadata and retrieval location). Each raster was developed at a cell size of 500 m. Three continuous RS were developed: topographic wetness index (TWI), elevation and slope. The Slope RS was developed based on our elevation raster by using the Slope (Spatial Analyst) tool in ArcGIS Pro and employing the geodesic method with the output measurement set to degrees. Then we altered the spatial resolution from 25 m to 500 m using the resampling tool and the bilinear resampling technique.

We used an IBR approach to assess the influence of landscape structure on *B. tryoni* population structure. We used the R package ResistanceGA v4.2–10 (Peterman 2018) to optimise RSs using CIRCUITSCAPE in Julia (Hall et al. 2021) using mean kinship (transformed to 1‐mean kinship) as the response variable, and an eight‐neighbour connection scheme. ResistanceGA uses a genetic algorithm to iteratively perform model optimisation until model support does not improve across 25 subsequent iterations. We conducted this as a two‐step analysis: the first is a univariate analysis with each RS optimised in isolation, the second is a multivariate analysis where the top performing univariate model is optimised simultaneously with an additional RS to create a composite RS. Model performance was ranked using Akaike Information Criterion corrected for finite sample size (AICc). The R package qpcR (Ritz and Spiess 2008) was used to calculate Akaike weight (*ω*) for each model.

### 2.5 Landscape Ecology

We used the Empirical Bayesian Kriging (Geostatistical Analyst Tools) tool implemented in ArcGIS Pro to interpolate *B. tryoni* abundance across the study area. We performed a Tukey HSD (Miller 1981; Yandell 1997) in R to determine whether the abundance of *B. tryoni* varied between land use types and across sampling periods. Additionally, we used linear mixed modelling (LMM) to investigate the top environmental predictors of *B. tryoni* abundance. The LMM analysis incorporated land use category information contained in the spatial layers retrieved from Qspatial, and these were pooled into five categories: grazing area, residential area, *Eucalyptus* woodland, rainforest and agricultural production, and the percentage area covered for each land use type within a 1 km radius of each site was calculated. Other variables included the distance from each site to the nearest perennial water source, and the latitude and longitude of each site. Latitude and longitude were included to test for potential population‐level effects of AWM. Specifically, we hypothesised that fly populations should be higher in and around the Bundaberg production area (in the north‐east of our sampling area) than the Gayndah/Mundubbera area (in the south‐west of our sampling area) (Figure 1) because of the absence or presence of AWM, respectively. Queensland fruit fly abundance was converted to catch per unit effort (CPUE) where the unit of effort was considered to be 1 day of trapping. The Tansey 1 site was omitted from the LMM analysis because the distance between Tansey 1 and Tansey 2 is only 1 km and would violate assumptions of spatial independence between sampling sites.

We used R package lme4 version 1.1–30 (Bates et al. 2015) and the R package MuMIn version 1.47.1 (Barton 2022) to assess all combinations of predictor variables, using sampling time as a random variable. The ‘dredge()’ function was used to rank all LMMs according to Akaike information criterion corrected for small sample size (AICc). Following dredging, the top performing LMMs according to AICc support were used to perform model averaging, implemented in the ‘model.avg.()’ function. This assessed each variable from the top performing models for their linear coefficient, standard error, statistical significance and upper and lower 95% confidence intervals.

## 3 Results

### 3.1 Population Structure

**FIGURE 2:**
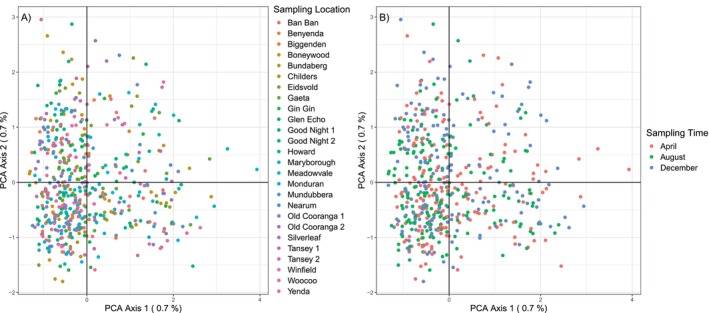
Principal coordinates analysis (PCoA) for 545 *Bactrocera tryoni* samples collected in April, August and December in the Wide Bay‐Burnett region of Southeast Queensland. Each plot displays the same PCoA with samples coloured according to (A) sampling site; (B) sampling month.

DArTseq produced a total of 53,368 SNPs, with 3759 SNPs retained after filtering for call rate (> 0.95), MAF (> 0.01) and deviations from HWE. Our resultant SNP dataset has a mean sequencing depth of 113× (std error ± 1.8), a minimum sequencing depth of 5×, and a maximum of 537×. H_O_ at each site across the three sampling periods ranged from 0.092 to 0.119, H_E_ ranged from 0.094 to 0.123, and F_IS_ values ranged from 0.005 to 0.136 (Appendix S2). Discriminant analysis of principal components (DAPC) indicated the optimal value of *K* = 1 (Appendix S3). FastStructure identified the optimal value for *K* to be within the range of *K* = 1–6 (model complexity that maximises marginal likelihood = 1 and model components used to explain structure in data = 6); however, the bar plots for the *K* = 2–6 scenarios did not provide a biologically meaningful interpretation of the genetic structure of *B. tryoni* (Appendix S4), indicating the samples derived from a single population with no detectable substructure. Principal coordinates analysis (PCoA) supported *K* = 1, as no clustering associated with site (Figure 2A) or time of collection (Figure 2B) was observed. The first two axes of the PCoA, PC1 and PC2, accounted for only 1.4% of total variation. The AMOVA also supports *K* = 1, with 99.96% of the variation found within populations and only 0.04% of variation among populations (Appendix S5). Estimates of N_e_ were infinite for all sampling sites at each sampling period. Only Childers in December had a noninfinite lower confidence interval (LCI = 2.2; Appendix S6). Estimates of N_e_ remain high when considering all time periods simultaneously; only Bundaberg has a noninfinite N_e_ (N_e_ = 706.8), and only Howard has a noninfinite lower confidence interval (LCI = 11,411.6). All upper confidence intervals are infinite.

Mean pairwise kinship values between sites ranged from 0.002 to 0.008 Appendix S7 and within sites ranged from 0.000 to 0.007 (Appendix S7) indicating no significant genetic structure across the study area or sampling times. Pairwise kinship values between individuals ranged from 0.000 to 0.146 (Appendix S7) indicating the presence of putative half‐siblings within the data set. There are six putative half‐sibling sets, each of which pertains to a comparison of individuals from different sampling sites, but within the same sampling period. The Mantel test found a statistically significant, but weak correlation between geographic distance and kinship during August (Appendices S8 and S9).

### 3.2 Landscape Genetics

**FIGURE 3:**
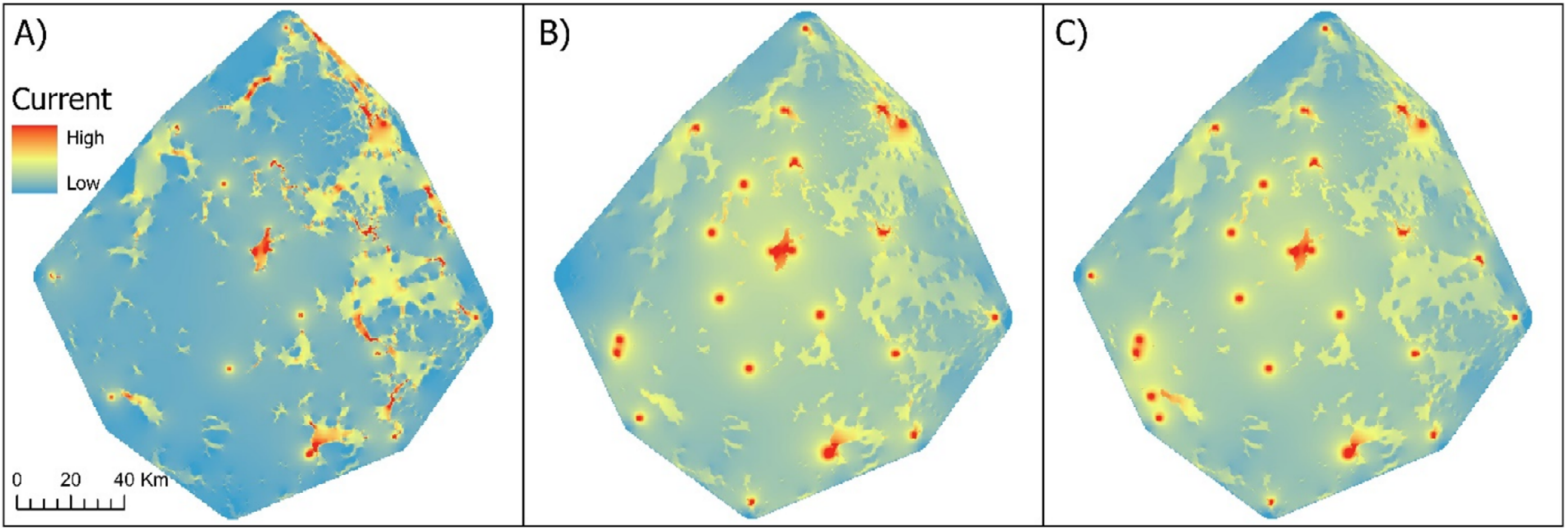
Current map of the Wide‐Bay/Burnett area representing the current density among sampling sites from (A) April, (B) August and (C) all time periods combined, based on the corresponding optimised grazing univariate resistance surface (RS). Higher currents indicate higher probabilities for random walkers to traverse that cell (McRae et al. 2008).

Landscape‐genetic analysis weakly supported several different models as predictors of *B. tryoni* gene flow across the study area. The top performing predictor for April was grazing area—the presence of grazing area was assigned an optimised resistance value of 21.97 (Appendix S10), indicating it is a barrier to gene flow. The top performing model in August was residential areas, though the distance‐only model was similarly supported (Table 1) corresponding with results from the Mantel test for this sampling period. Several other models, including the null model of no geographic structure, had an ∆AICc < 2 in August, indicating some support for alternative models as well. However, the optimised resistance values for all the categorical RSs in August are very low, indicating that the resistance imposed by the presence/absence of each modelled feature has little impact on gene flow. The top performing univariate model for all time periods combined is the distance‐only model, though the null model shows similar albeit lower support (Appendix S10). This is also supported by the mantel test for all time periods (*p* = 0.07; Appendices S8 and S9). The only two models with ∆AICc < 2 in the univariate analysis across April, August, and all sampling periods combined were the null model of no geographic structure and the grazing areas model; additionally, the optimised grazing areas model was always a barrier to gene flow, indicating *B. tryoni* gene flow is higher in the absence of grazing areas. Current maps generated based on the optimised grazing areas RS for each time period indicate potentially higher rates of gene flow across the eastern side of the study area (Figure 3). The grazing area RS was the only univariate model incorporated in all multivariate composite RSs due to inconsistent support for other univariate RSs. Only the TWI univariate RS was outperformed by any multivariate models in April; no multivariate models outperformed any univariate models in August, and the top performing model in the all‐time periods analysis is the multivariate surface of grazing area and highways (Table 1). The optimised resistance values for grazing area and highways identified grazing area (resistance = 56.49) and highways (resistance = 500.48) as strong barriers to gene flow. In this composite RS, the relative contribution of the grazing area (relative contribution = 87%) univariate model is considerably higher than the highways (relative contribution = 13%) univariate model. No other multivariate RSs in the all‐time periods analysis outperformed any univariate model. No landscape model could be determined for December due to a negative correlation between the genetic response variable and geographic distance. Due to the generally weak support shown for distance‐based models in both our Mantel tests and IBR analysis, we suspect the negative correlation between kinship and distance in December is a statistical artefact.

**TABLE 1:** Support for univariate and multivariate landscape resistance surfaces (RS) ranked by Akaike information criterion corrected for finite sample size (∆AICc) for *Bactrocera tryoni* populations collected from 26 sites across the Wide‐Bay Burnett region of Southeast Queensland for two sampling periods, plus all time periods which combine data for April, August and December samplings.

	Model	∆AICc	*ω*	*K*
April	Grazing	0.00	0.249	3
	Null	−0.20	0.226	1
	Slope	−1.31	0.130	4
	Distance	−2.83	0.061	2
	Residential areas	−3.07	0.054	3
	Highways and tracks	−4.02	0.033	3
	Elevation	−4.08	0.032	4
	Horticulture	−4.11	0.032	3
	Waterways	−4.19	0.031	3
	Highways	−4.35	0.028	3
	Eucalyptus	−4.37	0.028	3
	Rainforest	−4.47	0.027	3
	Grazing + rainforest	−6.43	0.010	5
	Grazing + highways	−6.45	0.010	5
	Grazing + horticulture	−6.81	0.008	5
	Grazing + waterways	−6.81	0.008	5
	Grazing + *Eucalyptus*	−6.95	0.008	5
	Grazing + highways and tracks	−6.96	0.008	5
	Grazing + residential	−7.00	0.008	5
	TWI	−8.21	0.004	4
	Grazing + slope	−8.29	0.004	6
	Grazing + elevation	−11.09	0.001	6
	Grazing + TWI	−11.73	0.001	6
August	Residential areas	0.00	0.176	3
	Distance	−0.16	0.162	2
	Null	−1.06	0.103	1
	Highways	−1.08	0.102	3
	Horticulture	−1.32	0.091	3
	Grazing	−1.43	0.086	3
	Highways & tracks	−1.73	0.074	3
	*Eucalyptus*	−2.21	0.058	3
	Waterways	−2.76	0.044	3
	Rainforest	−2.77	0.044	3
	Elevation	−4.92	0.015	4
	TWI	−5.52	0.011	4
	Slope	−5.55	0.011	4
	Grazing + horticulture	−7.33	0.004	5
	Grazing + waterways	−7.33	0.004	5
	Grazing + residential	−7.50	0.004	5
	Grazing + highways	−8.34	0.003	5
	Grazing + *Eucalyptus*	−8.74	0.002	5
	Grazing + rainforest	−8.89	0.002	5
	Grazing + highways and tracks	−8.91	0.002	5
	Grazing + TWI	−12.06	0.000	6
	Grazing + slope	−12.56	0.000	6
	Grazing + elevation	−12.67	0.000	6
All time periods	Grazing + highways	0.00	0.751	5
	Distance	−5.63	0.045	2
	Null	−6.17	0.034	1
	Horticulture	−6.19	0.034	3
	Highways	−6.48	0.029	3
	Grazing	−7.60	0.017	3
	Highways and tracks	−7.91	0.014	3
	Rainforest	−8.15	0.013	3
	Waterways	−8.17	0.013	3
	*Eucalyptus*	−8.20	0.012	3
	Residential	−8.20	0.012	3
	Elevation	−9.51	0.006	4
	Slope	−10.47	0.004	4
	TWI	−11.03	0.003	4
	Grazing + residential	−11.17	0.003	5
	Grazing + highways and tracks	−11.41	0.003	5
	Grazing + waterways	−12.10	0.002	5
	Grazing + horticulture	−12.14	0.002	5
	Grazing + rainforest	−13.44	0.001	5
	Grazing + *Eucalyptus*	−13.88	0.001	5
	Grazing + elevation	−16.33	0.000	6
	Grazing + slope	−16.53	0.000	6
	Grazing + TWI	−16.92	0.000	6

*Note:* December is not presented as an individual month due to a negative correlation between the genetic response variable and geographic distance. Also reported are the model weight (*ω*) and number of parameters (*K*) in each model. Results for the univariate only analysis are in Appendix S10.

### 3.3 Landscape Ecology

**FIGURE 4:**
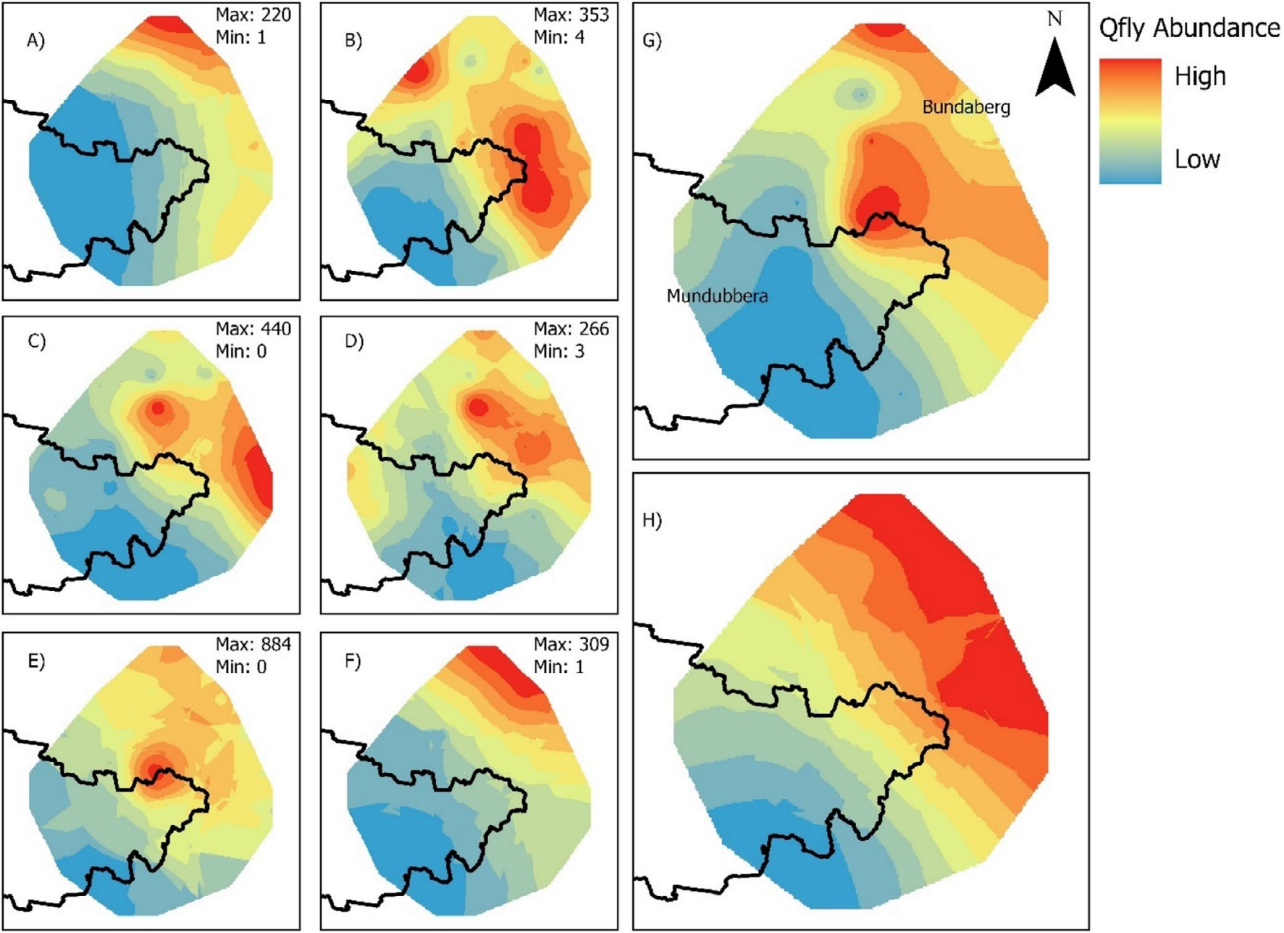
Empirical Bayesian kriging of *Bactrocera tryoni* abundance during each sampling period and map of fruit fly area‐wide management (AWM) boundary. (A) April 2021, (B) August 2021, (C) October 2021, (D) December 2021, (E) February 2022, (F) April 2022, (G) Average across all time periods, (H) Average abundance across all time periods excluding outlier from Good Night 2 in February 2022.

**FIGURE 5:**
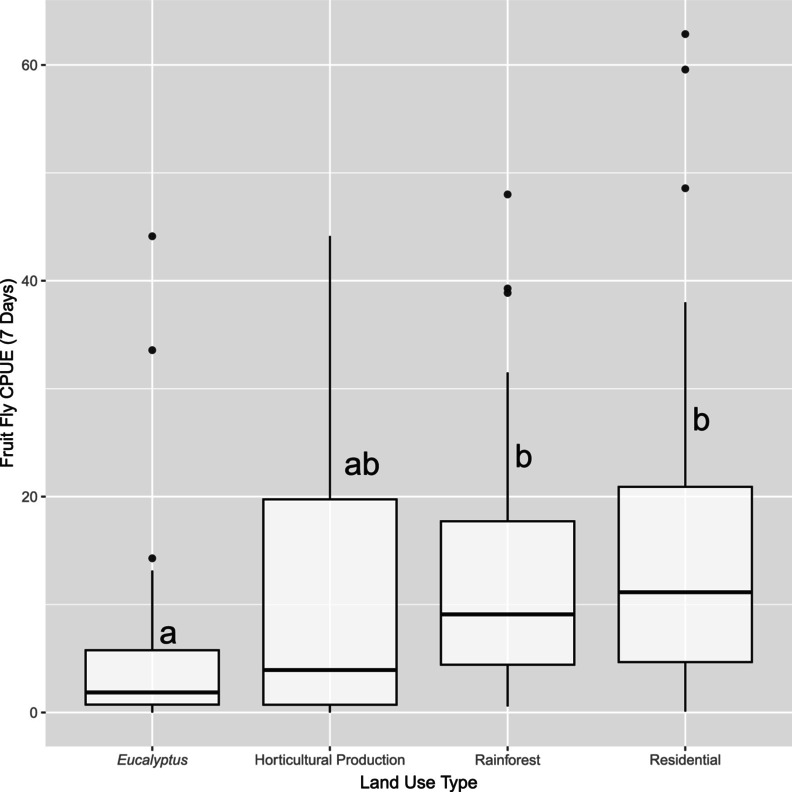
Box‐and‐whisker plots of *Bactrocera tryoni* abundance (catch per unit effort (CPUE)) over a 7‐day period at each sampling site for all time periods combined for each land use type (excludes outlier site Good Night 2 February 2022). Significant Tuley HSD pairwise comparisons are noted above each boxplot.

A total of 13,583 *B. tryoni* were caught during this study. Empirical Bayesian kriging of *B. tryoni* abundance for all time periods demonstrates a cline of high abundance in the north‐east to low abundance in the south‐west of the study area (Figure 4 and Appendix S11). This pattern is evident across all six sampling periods, though with minor fluctuations in the locations with the highest abundance. The site Good Night 2 in February 2022 is an outlier with a catch per unit effort of 126.3. The second highest catch per unit effort was less than half that value, at 62.9 for Gin Gin in October 2021. Tukey HSD tests detected a significant difference (*p* < 0.05) between the number of flies caught in *Eucalyptus* forest and both rainforest and residential areas (Appendices S12 and S13): Furthermore, when the outlier catch at Good Night 2 was removed the statistically significant differences between fruit fly abundance and catch habitat did not change (Figure 5 and Appendix S14). No other pairwise comparison was significantly different (*p* > 0.05). Subsequent analyses in the results omit the outlier site of Good Night 2, February 2022.

The LMM analysis included a total of 512 combinations of landscape predictors. Model averaging was performed using LMMs with ∆AICc < 2 from the top model (Table 2). The best models retained five predictors (Table 3) from the nine included in the analysis. The top performing model was a combination of grazing area and distance from the nearest water source with both negatively correlated with *B. tryoni* abundance. The second ranked model was a univariate model considering only grazing area, which had a negative correlation with *B. tryoni* abundance. The models ranked third, fourth and fifth included different combinations of grazing area and an additional variable (latitude, longitude and elevation). Following model averaging, grazing area was the only statistically significant landscape predictor of *B. tryoni* abundance (Table 3) and was negatively correlated. These findings are consistent with the results of the landscape‐genetic analysis where grazing area was the top performing landscape derived RS.

**TABLE 2:** Top five performing linear mixed models (LMMs) correlating *Bactrocera tyoni* abundance with landscape predictors.

Model	∆AICc	*ω*	df
Grazing + watersource	0.00	0.22	5
Grazing	0.01	0.22	4
Grazing + latitude	0.45	0.18	5
Grazing + longitude	1.40	0.11	5
Grazing + watersource + latitude	1.56	0.10	6
Grazing + latitude + longitude	1.82	0.09	6
Grazing + watersource + elevation	1.91	0.08	6

*Note:* Models are ranked according to AIC corrected for finite sample size (∆AICc) and Akaike weight (*ω*). Degrees of freedom (df) for each model are also given.

**TABLE 3:** Results of model averaging for the top five performing linear mixed models (LMMs).

Variable	Coefficient	Std. error	*Z*	*p*	2.5% CI	97.5% CI
(Intercept)	−31.32	247.3	0.125	0.900	−520.9	458.3
Grazing	−0.132	0.388	3.352	**8 × 10** ^**−4**^	**−0.209**	**−0.055**
Watersource	−0.006	0.010	0.608	0.543	−0.035	0.006
Latitude	0.126	2.384	0.525	0.600	−2.181	9.082
Longitude	0.523	1.604	0.323	0.747	−2.775	8.063
Elevation	8 × 10^−4^	0.005	0.163	0.870	−0.019	0.038

*Note:* Reported are the linear coefficient, standard error (Std. error), Z‐score, *p* value, 2.5% and 97.5% confidence intervals (CI). Bold text denotes statistical significance in a model and CIs that do not overlap 0.

## 4 Discussion

Our combined analysis of the landscape‐ecology and landscape genetics of *B. tryoni* found novel associations between landscape variables and abundance or gene flow while also confirming known aspects of its biology. Areas of grazing land were negatively associated with *B. tryoni* abundance, which agrees with predictions of a landscape suitability model for *B. tryoni* built using expert opinion (van Klinken et al. 2019). However, that grazing land can act as a barrier to dispersal was previously unknown and has significant implications for management. That no geographic population structure can be detected in the endemic range of *B. tryoni* has been previously established (Popa‐Báez et al. 2020). However, the kinship‐based landscape relatedness method used here provided novel data on dispersal in a highly abundant species with high dispersal capacity. Finally, we discuss the potential impacts of AWM in reducing population abundance of *B. tryoni* , demonstrating the value of this approach for the management of pest insects. These points are developed further below under the sections of landscape genetics, landscape ecology and implications for pest management.

### 4.1 Landscape Genetics

Several studies have evaluated the genetic structure of *B. tryoni* over large (multistate to continental) spatial scales, but none have examined genetic relatedness in a horticultural mosaic at a landscape scale. While previous studies have detected population genetic structuring in southern Australia, where *B. tryoni* populations are recently invasive (Gilchrist et al. 2006; Gilchrist and Meats 2010; Blacket et al. 2017), population structuring within the endemic tropical and subtropical range of *B. tryoni* is almost entirely absent (Gilchrist et al. 2006; Cameron et al. 2010; Popa‐Báez et al. 2020; Parvizi et al. 2024). Overall, our fine‐scale genetic data within a small region of *B. tryoni* 's endemic range support the findings of this previous research, with limited genetic structure evident among densely sampled areas. However, our study is novel because by using a systematic sampling design across the whole landscape we have been able to identify grazing land as a barrier to gene flow in this pest species. Our study highlights the value of landscape resistance modelling for the detection of barriers or facilitators of gene flow even in species displaying limited genetic structure (e.g., Pavlacky Jr et al. 2009; Verba et al. 2023). Previously, the only barriers to gene flow had been found among disjunct invasive populations of *B. tryoni* in Alice Springs, Victoria, and on Pacific Islands (Popa‐Báez et al. 2020). Furthermore, our study also highlights the potential for landscape genomic approaches to better inform management of pest insect species through the application of AWM to limit gene flow and to exploit natural barriers in the application of strategic sterile insect technique (SIT) releases (Schmidt et al. 2018, 2023).

Devising management strategies for pest species often relies on our ability to track individual and population‐level movement across landscapes. Currently, the value of genetic data to directly track the movement of *B. tryoni* and other tephritids at a landscape level remains unclear. Microsatellites (Gilchrist and Meats 2010) and mitochondrial sequence data (Blacket et al. 2017) were sufficient to identify and track geographically close populations of *B. tryoni* at a southern invasion front where effective population sizes were small. However, for both *B. tryoni* and melon fly, *Zeugodacus cucurbitae* (Coquillet), genome‐wide SNP analysis has detected minimal genetic structuring in their endemic habitat ranges of eastern Australia (Popa‐Báez et al. 2020) and Southeast Asia (Dupuis et al. 2018), respectively, although invasive populations are identifiable. In this study, we found that effective population sizes of *B. tryoni* are extremely large in the native range, and this obscures the detection of fine‐scale population structure. However, landscape‐relatedness measures (Norman et al. 2017), using kinship values, have been shown to outperform other methods for detecting population structure (e.g., F_ST_ and clustering algorithms) when effective population sizes approached infinity, or when population structure is weak (Iacchei et al. 2013; Palsbøll et al. 2010). Therefore, we hypothesize that landscape‐relatedness approaches in the invasive range may be useful for directly tracking *B. tryoni* movement at a landscape level and help devise management for pest insects. Sampling time may also affect the outcomes of genetic investigations as various RSs demonstrated varying levels of support depending on the sampling time. This may be artifactual given the very weak genetic structuring exhibited across all time periods. Alternatively, the capacity to detect genetic structure (if present) may be enhanced prior to intersubpopulation movement when more closely related individuals are in closer geographic proximity, that is, if sampling is conducted post‐winter, when *B. tryoni* population numbers begin to increase (Clarke et al. 2022). Our results show some support for this, with August (the earliest month of the year when the population numbers begin to increase) showing stronger support for IBD than April.

Highways emerged as a possible barrier to gene flow in our multivariate analysis. It is possible this barrier only emerges in the context of grazing area because highways are an extreme form of cleared land. However, simulation studies have shown that when high levels of ‘noise’ are introduced into the genetic response variable, multivariate analysis can have a high type I error rate (Winiarski et al. 2020). Due to our observed weak genetic structure, our data may have sufficient ‘noise’ for the MLPE to overfit the all‐time periods grazing area and highways composite model. If this pattern is not a type I error, the high relative importance of grazing area in the composite model further supports its role as a barrier to gene flow in our study.

### 4.2 Landscape Ecology

Significant expert opinion exists about the landscape ecology of *B. tryoni* , and this was captured in a landscape risk model by van Klinken et al. (2019). That expert opinion believes flies will be located in sheltered, humid sites with a good access to fruit for breeding. However, other than the current study, only Fletcher (1974a, 1974b) in temperate Australia and Raghu et al. (2000) in subtropical Australia have simultaneously sampled multiple habitat types so as to be able to directly compare habitat usage. Fletcher (1974b) and Raghu et al. (2000), and as modelled by van Klinken et al. (2019) and Schwarzmueller et al. 2019, found *B. tryoni* abundance high in urban sites. The near year‐round supply of fruit in urban backyards and their frequent watering resulting in high local humidity is thought to explain this pattern (Dominiak et al. 2006; Schwarzmueller et al. 2019). However, while we found *B. tryoni* was numerically most abundant in urban traps, it was not significantly more so than rainforest and horticultural production sites. While the importance of horticultural areas for *B. tryoni* breeding is self‐evident from its pest status, the importance of rainforest as breeding and sheltering sites for *B. tryoni* has been less clear, despite rainforest being considered the primary habitat type for Australian *Bactrocera* species (Starkie et al. 2024). Raghu et al. (2000) found *B. tryoni* to be rare in rainforest, while Drew et al. (1984) and our study collected large numbers of the fly from rainforest. There are numerous reasons why the results of the study may vary, but with two studies now capturing large numbers of *B. tryoni* in rainforest, it should be assumed that this is a suitable habitat type for the species as it is for other species in the genus. That *B. tryoni* can be trapped in eucalypt forest, even if in low numbers, is known from the literature (Raghu et al. 2000; Fletcher 1974a, 1974b), with Fletcher presenting both direct (1974a) and indirect evidence (1974b) that flies captured in eucalypt forest were dispersing through forest to and from breeding areas. This earlier trapping data, in combination with our genetic data and trapping data, strongly infers that while eucalypt forest should not be regarded as permanent habitat for *B. tryoni* , it is not a barrier to movement. That open grazing land is a barrier to *B. tryoni* movement and is negatively associated with abundance has not been previously identified.

### 4.3 Implications for Pest Management

That AWM can suppress local pest populations is well documented (Hendrichs et al. 2007), as was the case for the successful AWM of *B. tryoni* in the Gayndah–Mundubbera region when it was first implemented (Lloyd et al. 2007, 2010). Nevertheless, the data presented here is the first for Australian horticulture demonstrating the potential landscape effect of AWM between two neighbouring production regions (Figure 4), one with now long‐term AWM in place and the other without. In the large coastal production area of Bundaberg and smaller areas to its south, fly populations are very high, while in the south‐west of the study area around Gayndah and Mundubbera where AWM is implemented, fly populations are significantly lower. The AWM programme for Gayndah–Mundubbera is relatively simple, with fruit flies managed on‐farm and in rural towns (managing fruit on street trees, garden trees, etc.), but not in other favourable natural habitats such as rainforest (Lloyd et al. 2010). Existing wisdom suggests AWM is most likely to succeed where populations are relatively low, there are few uncontrolled breeding sites (i.e., no rainforest), and there is seasonally low temperature to break the breeding cycle (Jessup et al. 2007). In contrast, our data suggest that on‐farm and rural‐town management alone is sufficient to suppress *B. tryoni* populations regionally across a complex subtropical landscape where large populations and suitable temperatures occur year‐round, offering new opportunities for *B. tryoni* AWM in its native range.

A second and important aspect of our work for pest management is the fact that grazing land acts as a barrier to fruit fly dispersal and are negatively correlated with fruit fly abundance. The International Standards for Phytosanitary Measures (ISPMs) lay out internationally accepted guidelines for managing the phytosanitary risk posed by the movement or trade of plant and plant‐derived products. ISPM 26 (2006) and ISPM 35 (2012) deal with risk reduction processes for fruit fly impacted produce through the establishment of pest‐free areas or the implementation of systems approaches (a sequential series of independent risk reduction steps), respectively. For both approaches, knowing that open grazing lands are an impediment to *B. tryoni* movement is important. Very large areas of sheep and grazing country are common in Australia and production areas, or individual places of horticultural production (e.g., large glasshouses complexes), are commonly situated within surrounding grazing country. Acting as a deterrent to natural fruit fly movement and abundance, grazing lands fit with ISPM 26 Section 2.2.1 as contributing to ‘establishment of a buffer zone’ and within ISPM 35 Section 2 ‘selection of sites with low pest prevalence’.

## 5 Conclusion

Overall, this study demonstrates that both AWM and grazing land can reduce the abundance, and in the case of grazing land (and in composite with highways), gene flow in a pest insect. This finding reinforces previous research findings but also establishes the importance of carefully planned sampling regimes to test specific landscape‐genetic and ecological hypotheses in pest insects across horticultural landscape mosaics. Future work should continue to examine the potential of landscape‐related approaches to track outbreaks and incursions at range margins in mobile pest insects.

## Conflicts of Interest

The authors declare no conflicts of interest.

## Supporting information


Appendices S1–S14.

## Data Availability

Genetic, ecological and spatial data for this study are available through Open Science Framework at: https://osf.io/w4fk5/. DOI: 10.17605/OSF.IO/W4FK5.
