# Supplementary material for: Gene Flow and Abundance of a Tropical Fruit Fly in a Horticultural Landscape Mosaic in Eastern Australia Is Limited by Cleared Grazing Land and Area‐Wide Management
Source: Evol Appl. 2025 Apr 10;18(4):e70097. doi: 10.1111/eva.70097 (PMC11982624; doi:10.1111/eva.70097)
Supplement: Supplementary file 1 — Appendices S1–S14. [file EVA-18-e70097-s001.docx]

**Supporting Data**

Appendix 1 – Layer information including the name of the spatial layer retrieved from Qspatial, the corresponding resistance surface (RS) developed, and the type of RS developed. In this study the term “tracks” has been used to describe publicly assessable roadways which are not highways or main roads.

| Layer same (Source) | Resistance Surface | Type |
| --- | --- | --- |
| Baseline roads and tracks (Qspatial; <https://qldspatial.information.qld.gov.au/catalogue/custom/index.page>) | Highways | Categorical |
| Baseline roads and tracks (Qspatial) | Highways & tracks | Categorical |
| Digital elevation model 25metre – South East Queensland – data package (Qspatial) & Digital elevation model 25metre – Burnett catchment – data package (Qspatial) | Elevation | Continuous |
| Land use mapping 1999 – Current – Queensland (Qspatial) | Grazing | Categorical |
| Major watercourse lines – Queensland (Qspatial) & Watercourse areas – Queensland (Qspatial) | Waterways | Categorical |
| Remnant 2019 broad vegetation groups – Queensland (Qspatial) | *Eucalyptus* Woodland | Categorical |
| Remnant 2019 broad vegetation groups – Queensland (Qspatial) | Rainforest | Categorical |
| land use mapping 1999 – Current – Queensland (Qspatial) | Residential Areas | Categorical |
| Topographic Wetness Index from 1” SRTM DEM-H (CSIRO; https://data.csiro.au/collection/csiro:5588) | Topographic Wetness Index | Continuous |

Appendix 2 – Summary statistics for genetic analysis of each sampling site during each sampling period including number of samples (N), observed heterozygosity (H_O_), expected heterozygosity (H_e_) and inbreeding coefficient (F_IS_).

| Sampling Period | Pop | N | H_O_ | H_e_ | F_IS_ |
| --- | --- | --- | --- | --- | --- |
| April | Ban Ban | 5 | 0.09587 | 0.10966 | 0.12577 |
|  | Benyenda | 2 | 0.1079 | 0.11318 | 0.04664 |
|  | Biggenden | 7 | 0.10134 | 0.11373 | 0.10891 |
|  | Boneywood | 1 | 0.11249 | 0.11249 | NA |
|  | Bundaberg | 8 | 0.10104 | 0.11604 | 0.12925 |
|  | Childers | 11 | 0.10399 | 0.11005 | 0.05508 |
|  | Eidsvold | 9 | 0.10462 | 0.11694 | 0.10539 |
|  | Gaeta | 10 | 0.10863 | 0.11526 | 0.05747 |
|  | Gin Gin | 9 | 0.09936 | 0.1123 | 0.11524 |
|  | Glen Echo | 8 | 0.09959 | 0.11162 | 0.10777 |
|  | Good Night 1 | 10 | 0.09632 | 0.10747 | 0.10373 |
|  | Good Night 2 | 8 | 0.10148 | 0.1145 | 0.11369 |
|  | Howard | 12 | 0.11848 | 0.11957 | 0.00916 |
|  | Maryborough | 14 | 0.10186 | 0.11078 | 0.08054 |
|  | Meadowvale | 10 | 0.10005 | 0.1128 | 0.11299 |
|  | Monduran | 1 | 0.09733 | 0.09733 | NA |
|  | Mundubbera | 1 | 0.09492 | 0.09492 | NA |
|  | Nearum | 9 | 0.103 | 0.11502 | 0.1045 |
|  | Old Cooranga 1 | 9 | 0.09928 | 0.11176 | 0.11166 |
|  | Old Cooranga 2 | 1 | 0.10199 | 0.10199 | NA |
|  | Silverleaf | 4 | 0.10045 | 0.11305 | 0.11144 |
|  | Tansey 1 | 11 | 0.10672 | 0.11167 | 0.04429 |
|  | Tansey 2 | 7 | 0.09504 | 0.10928 | 0.13027 |
|  | Winfield | 7 | 0.10652 | 0.11406 | 0.06606 |
|  | Woocoo | 10 | 0.10374 | 0.11415 | 0.0912 |
|  | Yenda | 4 | 0.11991 | 0.12347 | 0.02882 |
| August | Ban Ban | 8 | 0.10089 | 0.1124 | 0.10238 |
|  | Benyenda | 8 | 0.10721 | 0.11773 | 0.08934 |
|  | Biggenden | 7 | 0.10036 | 0.11266 | 0.10919 |
|  | Boneywood | 8 | 0.09891 | 0.1109 | 0.10813 |
|  | Bundaberg | 7 | 0.09476 | 0.10699 | 0.11433 |
|  | Childers | 7 | 0.10096 | 0.11052 | 0.08651 |
|  | Eidsvold | 4 | 0.09222 | 0.10802 | 0.14625 |
|  | Gaeta | 7 | 0.09869 | 0.11192 | 0.11815 |
|  | Gin Gin | 8 | 0.0916 | 0.10607 | 0.13641 |
|  | Glen Echo | 8 | 0.09676 | 0.11113 | 0.12931 |
|  | Good Night 1 | 8 | 0.09911 | 0.11332 | 0.12543 |
|  | Good Night 2 | 6 | 0.09588 | 0.11017 | 0.12972 |
|  | Howard | 4 | 0.12171 | 0.12232 | 0.00498 |
|  | Maryborough | 8 | 0.10012 | 0.11033 | 0.09248 |
|  | Meadowvale | 7 | 0.10385 | 0.11384 | 0.08775 |
|  | Monduran | 7 | 0.10045 | 0.11175 | 0.10109 |
|  | Mundubbera | 8 | 0.09827 | 0.11004 | 0.10694 |
|  | Nearum | 7 | 0.10132 | 0.11121 | 0.08896 |
|  | Old Cooranga 1 | 3 | 0.09887 | 0.1121 | 0.11797 |
|  | Old Cooranga 2 | 7 | 0.11003 | 0.11773 | 0.06542 |
|  | Silverleaf | 8 | 0.10079 | 0.11128 | 0.09427 |
|  | Tansey 1 | 8 | 0.10779 | 0.11696 | 0.07841 |
|  | Tansey 2 | 7 | 0.09852 | 0.11172 | 0.1182 |
|  | Winfield | 7 | 0.10123 | 0.11268 | 0.10163 |
|  | Woocoo | 8 | 0.09366 | 0.10821 | 0.13445 |
|  | Yenda | 7 | 0.09603 | 0.10803 | 0.11103 |
| December | Ban Ban | 2 | 0.09856 | 0.11079 | 0.11036 |
|  | Benyenda | 4 | 0.09475 | 0.11054 | 0.1428 |
|  | Biggenden | 7 | 0.10318 | 0.11442 | 0.09822 |
|  | Boneywood | 8 | 0.09917 | 0.11369 | 0.12768 |
|  | Bundaberg | 8 | 0.11189 | 0.11978 | 0.06589 |
|  | Childers | 7 | 0.0984 | 0.11047 | 0.10923 |
|  | Eidsvold | 8 | 0.11822 | 0.12326 | 0.04091 |
|  | Gaeta | 6 | 0.09914 | 0.11209 | 0.11549 |
|  | Gin Gin | 7 | 0.09598 | 0.10922 | 0.12125 |
|  | Glen Echo | 8 | 0.10183 | 0.11156 | 0.08719 |
|  | Good Night 1 | 7 | 0.10196 | 0.1122 | 0.09128 |
|  | Good Night 2 | 8 | 0.10051 | 0.11176 | 0.10065 |
|  | Howard | 8 | 0.10785 | 0.11836 | 0.08882 |
|  | Maryborough | 8 | 0.09961 | 0.11216 | 0.11189 |
|  | Meadowvale | 8 | 0.10684 | 0.11582 | 0.07753 |
|  | Monduran | 8 | 0.10131 | 0.11318 | 0.10488 |
|  | Mundubbera | 9 | 0.10034 | 0.1144 | 0.12291 |
|  | Nearum | 8 | 0.10119 | 0.1132 | 0.10609 |
|  | Old Cooranga 1 | 8 | 0.09469 | 0.10872 | 0.12909 |
|  | Old Cooranga 2 | 5 | 0.09687 | 0.10952 | 0.11548 |
|  | Silverleaf | 8 | 0.10432 | 0.11664 | 0.10563 |
|  | Tansey 1 | 9 | 0.107 | 0.11673 | 0.08335 |
|  | Tansey 2 | 5 | 0.09944 | 0.11309 | 0.12067 |
|  | Winfield | 9 | 0.1035 | 0.11722 | 0.11698 |
|  | Woocoo | 1 | 0.0941 | 0.0941 | NA |
|  | Yenda | 1 | 0.10196 | 0.10196 | NA |

Appendix 3 – Bayesian Information Criterion (BIC) values for values of K from 1 – 20 determined using the find.clusters() function in the adegenet R package. According to BIC the optimal value is K = 1.


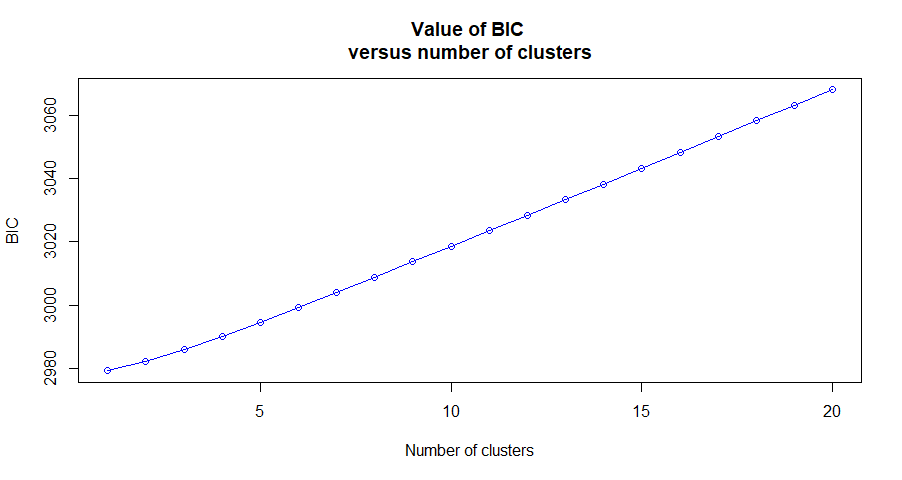


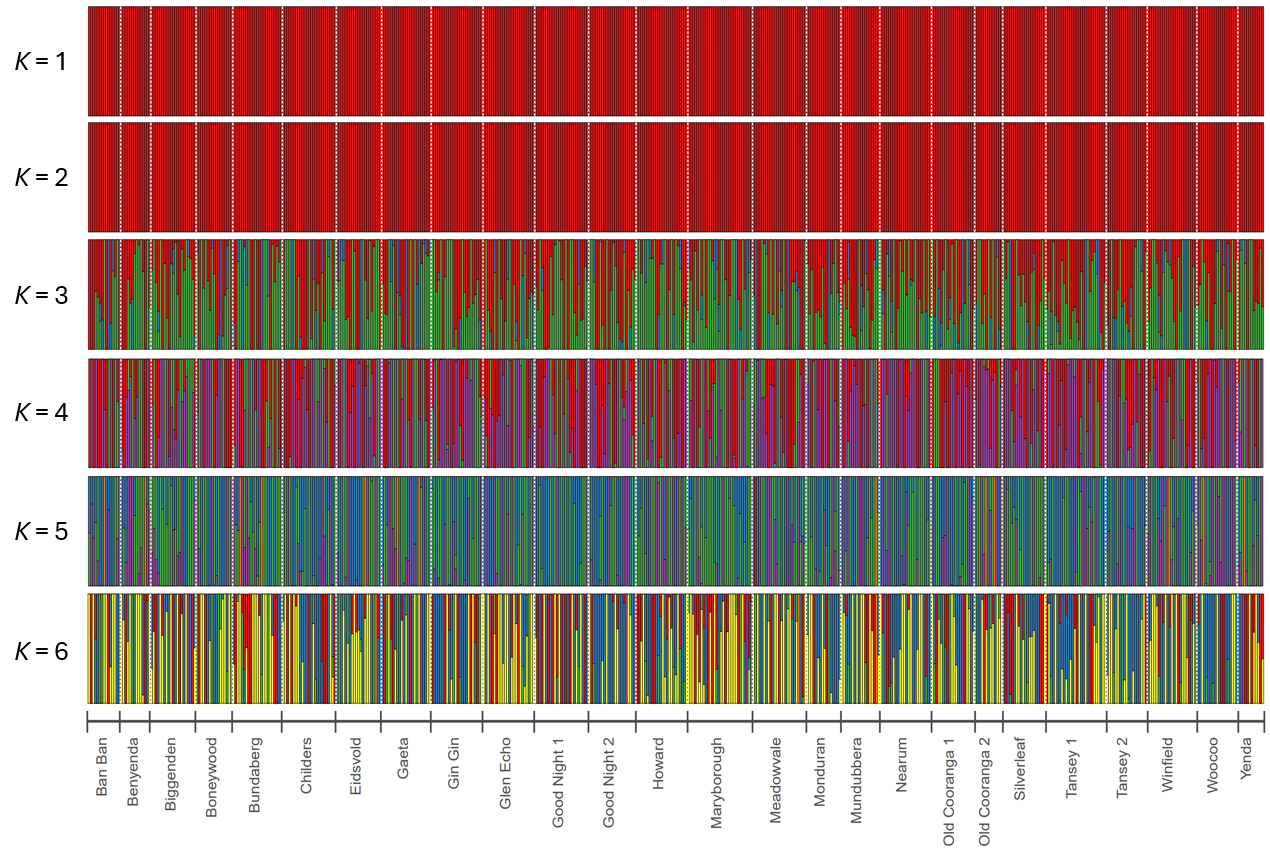


Appendix 4 – Barplots showing the Q assignment for each *Bactrocera tryoni* under each possible *K* scenario (*K* = 1 – 6) based on results of the fastStructure clustering algorithm. The *K* = 2 barplot shows > 99% Q-assignment to a single cluster for all individuals and is largely indistinguishable from *K* = 1.

Appendix 5 – Results of the AMOVA showing the % genetic variation stored between populations and within populations. Degrees of Freedom (Df).

|  | Df | Sum of Squares | Mean of Squares | σ | % Variation |
| --- | --- | --- | --- | --- | --- |
| Between Populations | 77 | 38.279 | 0.497 | 0.000 | 0.038 |
| Within Populations | 467 | 231.538 | 0.496 | 0.496 | 99.962 |
| Total | 544 | 269.816 | 0.496 | 0.496 | 100 |

Appendix 6 – Estimates of *Bactrocera tryoni* effective population size (N_e_) using the linkage disequilibrium method for 26 sites in the Wide-Bay Burnett region of South-east Queensland over three sampling periods. Results for a minor allele frequency (MAF) of 0.05 and 0.01 are reported and the upper and lower 95% confidence intervals for each MAF.

| Month | Pop | N | N_e_ | Lower CI | Upper CI |
| --- | --- | --- | --- | --- | --- |
| April | Ban Ban | 5 | ∞ | ∞ | ∞ |
|  | Benyenda | 2 | ∞ | ∞ | ∞ |
|  | Biggenden | 7 | ∞ | ∞ | ∞ |
|  | Boneywood | 1 | ∞ | ∞ | ∞ |
|  | Bundaberg | 8 | ∞ | ∞ | ∞ |
|  | Childers | 11 | ∞ | ∞ | ∞ |
|  | Eidsvold | 9 | ∞ | ∞ | ∞ |
|  | Gaeta | 10 | ∞ | ∞ | ∞ |
|  | Gin Gin | 9 | ∞ | ∞ | ∞ |
|  | Glen Echo | 8 | ∞ | ∞ | ∞ |
|  | Good Night 1 | 10 | ∞ | ∞ | ∞ |
|  | Good Night 2 | 8 | ∞ | ∞ | ∞ |
|  | Howard | 12 | ∞ | ∞ | ∞ |
|  | Maryborough | 14 | ∞ | ∞ | ∞ |
|  | Meadowvale | 10 | ∞ | ∞ | ∞ |
|  | Monduran | 1 | ∞ | ∞ | ∞ |
|  | Mundubbera | 1 | ∞ | ∞ | ∞ |
|  | Nearum | 9 | ∞ | ∞ | ∞ |
|  | Old Cooranga 1 | 9 | ∞ | ∞ | ∞ |
|  | Old Cooranga 2 | 1 | ∞ | ∞ | ∞ |
|  | Silverleaf | 4 | ∞ | ∞ | ∞ |
|  | Tansey 1 | 11 | ∞ | ∞ | ∞ |
|  | Tansey 2 | 7 | ∞ | ∞ | ∞ |
|  | Winfield | 7 | ∞ | ∞ | ∞ |
|  | Woocoo | 10 | ∞ | ∞ | ∞ |
|  | Yenda | 4 | ∞ | ∞ | ∞ |
| August | Ban Ban | 8 | ∞ | ∞ | ∞ |
|  | Benyenda | 8 | ∞ | ∞ | ∞ |
|  | Biggenden | 7 | ∞ | ∞ | ∞ |
|  | Boneywood | 8 | ∞ | ∞ | ∞ |
|  | Bundaberg | 7 | ∞ | ∞ | ∞ |
|  | Childers | 7 | ∞ | ∞ | ∞ |
|  | Eidsvold | 4 | ∞ | ∞ | ∞ |
|  | Gaeta | 7 | ∞ | ∞ | ∞ |
|  | Gin Gin | 8 | ∞ | ∞ | ∞ |
|  | Glen Echo | 8 | ∞ | ∞ | ∞ |
|  | Good Night 1 | 8 | ∞ | ∞ | ∞ |
|  | Good Night 2 | 6 | ∞ | ∞ | ∞ |
|  | Howard | 4 | ∞ | ∞ | ∞ |
|  | Maryborough | 8 | ∞ | ∞ | ∞ |
|  | Meadowvale | 7 | ∞ | ∞ | ∞ |
|  | Monduran | 7 | ∞ | ∞ | ∞ |
|  | Mundubbera | 8 | ∞ | ∞ | ∞ |
|  | Nearum | 7 | ∞ | ∞ | ∞ |
|  | Old Cooranga 1 | 3 | ∞ | ∞ | ∞ |
|  | Old Cooranga 2 | 7 | ∞ | ∞ | ∞ |
|  | Silverleaf | 8 | ∞ | ∞ | ∞ |
|  | Tansey 1 | 8 | ∞ | ∞ | ∞ |
|  | Tansey 2 | 7 | ∞ | ∞ | ∞ |
|  | Winfield | 7 | ∞ | ∞ | ∞ |
|  | Woocoo | 8 | ∞ | ∞ | ∞ |
|  | Yenda | 7 | ∞ | ∞ | ∞ |
| December | Ban Ban | 2 | ∞ | ∞ | ∞ |
|  | Benyenda | 4 | ∞ | ∞ | ∞ |
|  | Biggenden | 7 | ∞ | ∞ | ∞ |
|  | Boneywood | 8 | ∞ | ∞ | ∞ |
|  | Bundaberg | 8 | ∞ | ∞ | ∞ |
|  | Childers | 7 | ∞ | 2.2 | ∞ |
|  | Eidsvold | 8 | ∞ | ∞ | ∞ |
|  | Gaeta | 6 | ∞ | ∞ | ∞ |
|  | Gin Gin | 7 | ∞ | ∞ | ∞ |
|  | Glen Echo | 8 | ∞ | ∞ | ∞ |
|  | Good Night 1 | 7 | ∞ | ∞ | ∞ |
|  | Good Night 2 | 8 | ∞ | ∞ | ∞ |
|  | Howard | 8 | ∞ | ∞ | ∞ |
|  | Maryborough | 8 | ∞ | ∞ | ∞ |
|  | Meadowvale | 8 | ∞ | ∞ | ∞ |
|  | Monduran | 8 | ∞ | ∞ | ∞ |
|  | Mundubbera | 9 | ∞ | ∞ | ∞ |
|  | Nearum | 8 | ∞ | ∞ | ∞ |
|  | Old Cooranga 1 | 8 | ∞ | ∞ | ∞ |
|  | Old Cooranga 2 | 5 | ∞ | ∞ | ∞ |
|  | Silverleaf | 8 | ∞ | ∞ | ∞ |
|  | Tansey 1 | 9 | ∞ | ∞ | ∞ |
|  | Tansey 2 | 5 | ∞ | ∞ | ∞ |
|  | Winfield | 9 | ∞ | ∞ | ∞ |
|  | Woocoo | 1 | ∞ | ∞ | ∞ |
|  | Yenda | 1 | ∞ | ∞ | ∞ |
| ATP | Ban Ban | 15 | ∞ | ∞ | ∞ |
|  | Benyenda | 14 | ∞ | ∞ | ∞ |
|  | Biggenden | 21 | ∞ | ∞ | ∞ |
|  | Boneywood | 17 | 706.8 | ∞ | ∞ |
|  | Bundaberg | 23 | ∞ | ∞ | ∞ |
|  | Childers | 25 | ∞ | ∞ | ∞ |
|  | Eidsvold | 21 | ∞ | ∞ | ∞ |
|  | Gaeta | 23 | ∞ | ∞ | ∞ |
|  | Gin Gin | 24 | ∞ | ∞ | ∞ |
|  | Glen Echo | 24 | ∞ | ∞ | ∞ |
|  | Good Night 1 | 25 | ∞ | ∞ | ∞ |
|  | Good Night 2 | 22 | ∞ | ∞ | ∞ |
|  | Howard | 24 | ∞ | 11,411.6 | ∞ |
|  | Maryborough | 30 | ∞ | ∞ | ∞ |
|  | Meadowvale | 25 | ∞ | ∞ | ∞ |
|  | Monduran | 16 | ∞ | ∞ | ∞ |
|  | Mundubbera | 18 | ∞ | ∞ | ∞ |
|  | Nearum | 24 | ∞ | ∞ | ∞ |
|  | Old Cooranga 1 | 20 | ∞ | ∞ | ∞ |
|  | Old Cooranga 2 | 13 | ∞ | ∞ | ∞ |
|  | Silverleaf | 20 | ∞ | ∞ | ∞ |
|  | Tansey 1 | 28 | ∞ | ∞ | ∞ |
|  | Tansey 2 | 19 | ∞ | ∞ | ∞ |
|  | Winfield | 23 | ∞ | ∞ | ∞ |
|  | Woocoo | 19 | ∞ | ∞ | ∞ |
|  | Yenda | 12 | ∞ | ∞ | ∞ |

Appendix 8: Correlation coefficient (r) and p value of the Mantel Test for *Bactrocera tryoni* populations collected from 26 sites across the Wide-Bay Burnett region of South-east Queensland for each of three sampling periods according to geographic distance (m) compared to mean kinship (transformed to 1 – mean kinship).

| Month | r | p-value |
| --- | --- | --- |
| April | 0.0925 | 0.14 |
| August | **0.1430** | **0.01** |
| December | -0.0294 | 0.67 |
| All Time Periods | 0.0952 | 0.07 |


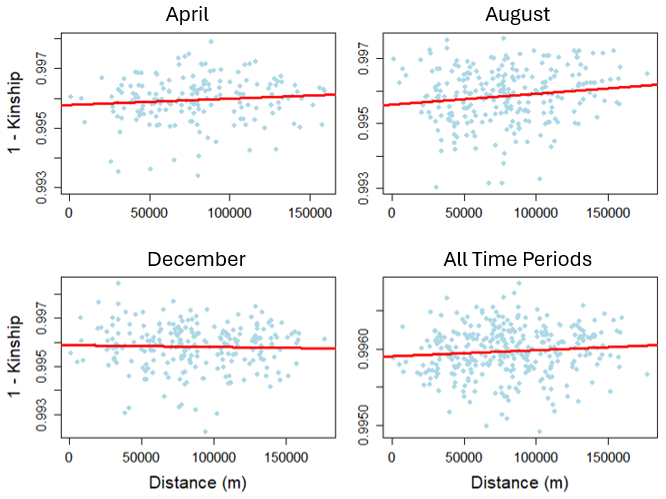


Appendix 9 – Scatterplots of the correlation between 1 – kinship and geographic distance (m) for all sites with ≥ 5 individuals.

Appendix 10: Support for univariate landscape resistance surfaces (RS) ranked by Akaike information criterion corrected for finite sample size (∆AICc) for *Bactrocera tryoni* populations collected from 26 sites across the Wide-Bay Burnett region of South-east Queensland for two sampling periods, plus all time periods which combines data for April, August and December samplings. December is not presented as an individual month due to a negative correlation between the genetic response variable and geographic distance. Akaike weight (ω) shows relative likelihood of each RS; Transformation differs between continuous and categorical RSs. For continuous RSs, “T” is the transformation ResistanceGA applies to during model optimisation and “Max” is the maximum resistance in the transformed RS. For categorical RSs “A” is the optimised resistance value for the absence or “P” presence of the landscape predictors in the original RS. Also reported are the number of parameters (K) in each model.

|  | Model | ∆AICc | ω | Transformation | | K |
| --- | --- | --- | --- | --- | --- | --- |
| April | Grazing | 0.00 | 0.26 | P:21.97 | A: 1 | 1 |
|  | Null | -0.20 | 0.23 | NA | | 3 |
|  | Slope | -1.31 | 0.13 | T: M | Max: 528.14 | 4 |
|  | Distance | -2.83 | 0.10 | NA | | 2 |
|  | Residential Areas | -3.07 | 0.06 | P: 1 | A: 5.71 | 3 |
|  | Highways & Tracks | -4.02 | 0.03 | P: 1 | A: 3.78 | 3 |
|  | Elevation | -4.08 | 0.03 | T: M | Max: 964.31 | 4 |
|  | Horticulture | -4.11 | 0.03 | P: 1 | A: 18.91 | 3 |
|  | Waterways | -4.19 | 0.03 | P:1 | A: 5.31 | 3 |
|  | Highways | -4.35 | 0.03 | P: 1 | A: 8.28 | 3 |
|  | *Eucalyptus* | -4.37 | 0.03 | P: 2.21 | A: 1 | 3 |
|  | Rainforest | -4.47 | 0.03 | P: 1.95 | A: 1 | 3 |
|  | TWI | -8.21 | 0.00 | T: IRM | Max: 48.92 | 4 |
| August | Residential Areas | 0.00 | 0.18 | P: 1 | A: 3.95 | 3 |
|  | Distance | -0.16 | 0.17 | NA | | 2 |
|  | Null | -1.06 | 0.11 | NA | | 1 |
|  | Highways | -1.08 | 0.10 | P: 1 | A: 4.54 | 3 |
|  | Horticulture | -1.32 | 0.09 | P: 2.80 | A: 1 | 3 |
|  | Grazing | -1.43 | 0.09 | P: 2.55 | A: 1 | 3 |
|  | Highways & Tracks | -1.73 | 0.08 | P: 1 | A: 3.79 | 3 |
|  | *Eucalyptus* | -2.21 | 0.06 | P: 1.63 | A: 1 | 3 |
|  | Waterways | -2.76 | 0.05 | P: 1 | A: 1.40 | 3 |
|  | Rainforest | -2.77 | 0.05 | P: 1.21 | A: 1 | 3 |
|  | Elevation | -4.92 | 0.02 | T: RR | Max: 996.26 | 4 |
|  | TWI | -5.52 | 0.01 | T: IRM | A: 105.21 | 4 |
|  | Slope | -5.55 | 0.01 | T: IRM | Max: 827.65 | 4 |
| All Time periods | Distance | 0.00 | 0.19 | NA | | 2 |
|  | Null | -0.54 | 0.14 | NA | | 1 |
|  | Horticulture | -0.56 | 0.14 | P: 1 | A: 289.80 | 3 |
|  | Highways | -0.85 | 0.12 | P: 7.69 | A: 1 | 3 |
|  | Grazing | -1.97 | 0.07 | P: 2.46 | A: 1 | 3 |
|  | Highways & Tracks | -2.28 | 0.06 | P: 2.05 | A: 1 | 3 |
|  | Rainforest | -2.52 | 0.05 | P: 1.28 | A: 1 | 3 |
|  | Waterways | -2.54 | 0.05 | P: 1 | A: 1.41 | 3 |
|  | *Eucalyptus* | -2.57 | 0.05 | P: 1.06 | A: 1 | 3 |
|  | Residential | -2.57 | 0.05 | P: 1.02 | A: 1 | 3 |
|  | Elevation | -3.88 | 0.03 | T: M | Max: 3.69 | 4 |
|  | Slope | -4.84 | 0.02 | T: M | Max: 1.43 | 4 |
|  | TWI | -5.40 | 0.01 | T: IRM | 883.84 | 4 |

Appendix 11 – *Bactrocera tryoni* catch per unit effort (CPUE) per sampling site per sampling period. CPUE is calculated based on the expected number of *B. tryoni* after seven days of sampling.

| Site | Landscape type | April 2021 CPUE | August 2021 CPUE | October 2021 CPUE | December 2021 CPUE | February 2022 CPUE | April 2022 CPUE | CPUE_sum |
| --- | --- | --- | --- | --- | --- | --- | --- | --- |
| Ban Ban | *Eucalyptus* | 0.64 | 3.38 | 2.00 | 0.43 | 2.00 | 0.29 | 8.73 |
| Benyanda | Agriculture | 0.18 | 1.50 | 0.00 | 0.57 | 0.00 | 0.14 | 2.40 |
| Biggenden | Residential | 2.36 | 24.13 | 21.43 | 18.00 | 7.43 | 1.29 | 74.63 |
| Boneywood | Agriculture | 0.09 | 2.38 | 3.43 | 15.86 | 9.71 | 0.71 | 32.18 |
| Bundaberg | Residential | 1.36 | 4.50 | 32.14 | 6.29 | 9.43 | 11.43 | 65.15 |
| Childers | Residential | 4.55 | 37.63 | 21.14 | 23.00 | 18.00 | 5.57 | 109.88 |
| Eidsvold | Residential | 1.00 | 21.00 | 10.86 | 19.86 | 9.57 | 6.86 | 69.14 |
| Gaeta | *Eucalyptus* | 1.82 | 44.13 | 13.14 | 7.86 | 14.29 | 4.71 | 85.94 |
| Gin Gin | Residential | 3.18 | 26.13 | 62.86 | 38.00 | 19.14 | 7.29 | 156.59 |
| Glen Echo | Rainforest | 9.09 | 27.75 | 6.00 | 4.43 | 20.71 | 11.71 | 79.70 |
| Good Night 1 | Rainforest | 2.09 | 31.50 | 24.29 | 6.29 | 48.00 | 17.71 | 129.88 |
| Good Night 2 | Rainforest | 1.91 | 15.13 | 39.29 | 13.29 | 126.29 | 8.86 | 204.75 |
| Howard | Residential | 8.73 | 20.63 | 48.57 | 16.71 | 17.29 | 10.29 | 122.21 |
| Maryborough | Residential | 2.73 | 20.88 | 59.57 | 18.00 | 9.86 | 4.71 | 115.75 |
| Meadowvale | Agriculture | 12.45 | 30.88 | 7.14 | 7.14 | 21.57 | 44.14 | 123.33 |
| Monduran | *Eucalyptus* | 0.55 | 4.38 | 0.43 | 2.86 | 7.57 | 1.43 | 17.21 |
| Mundubbera | Residential | 0.09 | 1.38 | 17.71 | 14.43 | 1.71 | 1.29 | 36.61 |
| Nearum | *Eucalyptus* | 1.18 | 12.88 | 33.57 | 5.57 | 8.00 | 2.86 | 64.06 |
| Old Cooranga 1 | *Eucalyptus* | 1.09 | 0.50 | NA | 7.57 | 1.57 | 0.86 | 11.59 |
| Old Cooranga 2 | *Eucalyptus* | 0.18 | 3.38 | NA | 1.86 | 1.86 | 1.86 | 9.13 |
| Silverleaf | Agriculture | 0.36 | 2.38 | 0.71 | 4.43 | 0.71 | 0.71 | 9.31 |
| Tansey 1 | Rainforest | 5.09 | 4.25 | 0.86 | 2.00 | 9.86 | 3.86 | 25.91 |
| Tansey 2 | Rainforest | 1.36 | 4.88 | 0.57 | 1.14 | 6.00 | 1.57 | 15.52 |
| Winfield | Agriculture | 20.00 | 19.00 | 32.57 | 20.86 | 37.86 | 37.86 | 168.14 |
| Woocoo | Rainforest | 5.45 | 38.88 | 11.86 | 0.57 | 16.00 | 11.86 | 84.62 |
| Yenda | *Eucalyptus* | 0.73 | 6.38 | 0.00 | 0.86 | 0.43 | 0.71 | 9.10 |
| Total | NA | **88.27** | **409.75** | **450.14** | **257.86** | **424.86** | **200.57** | NA |

Appendix 12 – Tukey HSD test of *Bactrocera tryoni* abundance for each landscape type including the outlier site at Good Night 2 February 2022.

| Landscape 1 | Landscape 2 | diff | lwr | upr | p |
| --- | --- | --- | --- | --- | --- |
| *Eucalyptus* | Rainforest | 12.005 | 2.176 | 21.833 | **0.010** |
| *Eucalyptus* | Residential | 10.480 | 1.768 | 19.192 | **0.011** |
| *Eucalyptus* | Horticultural production | 6.035 | -3.794 | 15.863 | 0.384 |
| Rainforest | Residential | -1.524 | -10.995 | 7.946 | 0.975 |
| Rainforest | Horticultural production | 5.970 | -4.537 | 16.477 | 0.454 |
| Residential | Horticultural production | 4.446 | -5.025 | 13.917 | 0.615 |


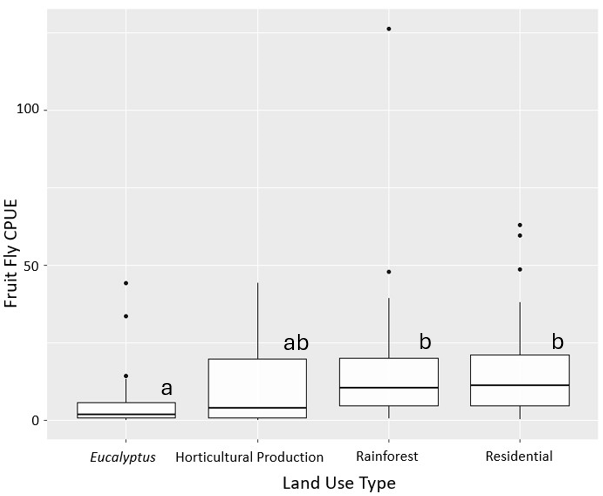


Appendix 13 – Boxplots and whisker plots of *Bactrocera tryoni* abundance (catch per unit effort (CPUE) over a seven-day period) at each sampling site across all time periods for each landscape type including the outlier site Good Night 2 February 2022. Significant Tuley HSD pairwise comparisons are noted above each boxplot.

Appendix 14 – Tukey HSD test of *Bactrocera tryoni* abundance for each land use type (excluding outlier site Good Night 2 February 2022).

| Category 1 | Category 2 | Difference | Lower | Upper | p-value |
| --- | --- | --- | --- | --- | --- |
| *Eucalyptus* | Rainforest | 8.242 | 0.206 | 16.277 | **0.042** |
| *Eucalyptus* | Residential | 10.480 | 3.427 | 17.534 | **0.001** |
| *Eucalyptus* | Horticultural Production | 6.035 | -1.923 | 13.993 | 0.204 |
| Rainforest | Residential | 2.239 | -5.510 | 9.988 | 0.876 |
| Rainforest | Horticultural Production | 2.207 | -6.373 | 10.787 | 0.909 |
| Residential | Horticultural Production | 4.446 | -3.223 | 12.114 | 0.436 |
